# The Effect of Microbial Endophyte Consortia on *Pseudotsuga menziesii* and *Thuja plicata* Survival, Growth, and Physiology Across Edaphic Gradients

**DOI:** 10.3389/fmicb.2019.01353

**Published:** 2019-06-18

**Authors:** Matthew M. Aghai, Zareen Khan, Matthew R. Joseph, Aubrey M. Stoda, Andrew W. Sher, Gregory J. Ettl, Sharon L. Doty

**Affiliations:** ^1^UW Center for Sustainable Forestry at Pack Forest, Eatonville, WA, United States; ^2^School of Environmental and Forest Sciences, University of Washington, Seattle, WA, United States

**Keywords:** biofertilizer, Douglas-fir (*Pseudotsuga menziesii*), drought, endophytes, microbiome, reforestation, stress mitigation, western redcedar (*Thuja plicata*)

## Abstract

Increased frequency of droughts and degraded edaphic conditions decreases the success of many reforestation efforts in the Pacific Northwest. Microbial endophyte consortia have been demonstrated to contribute to plant growth promotion and protection from abiotic and biotic stresses – specifically drought conditions – across a number of food crops but for limited tree species. Our research aimed to investigate the potential to improve establishment of economically and ecologically important conifers through a series of *in situ* field trials and *ex situ* simulations. Microbial endophyte consortia from Salicaceae, previously shown to confer drought tolerance, and conifer endophyte strains with potentially symbiotic traits were selected for trials with Douglas-fir (*Pseudotsuga menziesii*) and western redcedar (*Thuja plicata*). Reductive experimentation was used to subject seedlings to a spectrum of simulated drought levels or presence/absence of fertilizer, testing hypotheses that endophyte consortia impart improved drought resistance and growth promotion, respectively. Inoculation from Salicaceae consortia significantly (*p* ≤ 0.05) improved survival among seedlings of both species subject to increasing drought stress, with *T. plicata* seedlings surviving at twofold higher rates in extreme drought conditions. Both species demonstrated improved growth 540 days after inoculation of seed with conifer derived consortia. In the carefully controlled greenhouse experiments with both species, seedling Fv/Fm and SPAD values remained significantly (*p* ≤ 0.05) more stable in inoculated treatment groups as stress increased. Our findings confirm that multi-strain consortia may be applied as seed or field amendment to conifers, and the approach is efficient in garnering a positive growth response and can mitigate abiotic stressors.

## Introduction

Endophytes, microorganisms living within plants, are diverse, widely distributed, and essential in a range of ecological functions. The vast majority of endophytes are non-pathogenic and probably commensal with their hosts (Wilson, 2000; Mitter et al., 2013; Brader et al., 2017). Endophytes may influence shifts in biogeography and plant communities (Rodriguez et al., 2009; Bulgarelli et al., 2013; Sikes et al., 2016), contribute to nutrient cycling (Moyes et al., 2016), and enhance crop production (Le Cocq et al., 2017). Fungal and bacterial endophytes isolated as individual strains and consortia (i.e., communities of strains) have been subjected to reductive analyses to determine their role in enhancing plant growth in some agricultural species (Chanway, 1996; Mitter et al., 2013; Hardoim et al., 2015). Endophyte applications to food crops can improve survival, growth, yields, and pathogen resistance (Senthilkumar et al., 2011; Le Cocq et al., 2017; Rho et al., 2018a, b).

The role of endophytes in forest communities remains poorly understood; however, high-throughput technologies have increased the speed and resolution in screening microbial communities (Hardoim et al., 2015; Baldrian, 2017). Endophyte inoculated trees have shown: (1) positive growth trends (Chanway and Holl, 1991; Chanway et al., 1994; Shishido et al., 1999; Khan et al., 2015; Castro et al., 2017), (2) adaptive phenotypic changes (Knoth et al., 2014; Vivas et al., 2015), (3) stress mitigation (Khan et al., 2016; Doty, 2017; Doty et al., 2017; Rho et al., 2018a, b), (4) nitrogen fixation (Anand and Chanway, 2013; Anand et al., 2013; Knoth et al., 2014), and (5) reduced damage from insect herbivores and fungal pathogens (Brooks et al., 1994; Miller, 2011; Pirttilä and Frank, 2011).

A more contemporary understanding of plant health considers the plant holobiont (i.e., the host genome and its microbiome) as a co-evolved unit that is constantly adapting to the dynamic abiotic and biotic environment (Guerrero et al., 2013). Continued research of the holobiont will have implications on both ecosystem science (e.g., quantifying forest nitrogen budgets; Moyes et al., 2016) and management (e.g., developing more robust planting stock for restoration, phytoremediation, and biomass production; Anand et al., 2006; Ryan et al., 2008; Doty, 2017; Doty et al., 2017).

Artificial regeneration of conifers, specifically Douglas-fir (*Pseudotsuga menziesii*) and western redcedar (*Thuja plicata*), is ecologically and economically integral across western North America, and parts of Europe, New Zealand, South Africa, and Patagonia where they are naturalized or used in fiber plantations. Improved nursery and planting practices, including production of larger caliper planting stock, introduction of fertilizers, beneficial mycorrhizal inoculants, and weed control have increased field survival of planted seedlings above 80% (Sinclair et al., 1982; Molina and Trappe, 1984; Talbert and Marshall, 2005). Improved understanding of the microbiome may enhance nursery culture and facilitate better plant establishment following outplanting by increasing the availability of essential nutrients, conferring pathogen resistance, improving fitness, and enhancing ecophysiological function in response to abiotic and biotic stressors (Glick et al., 1997; Rodriguez et al., 2009; Aroca, 2012; Hardoim et al., 2015).

Endophyte-inoculated trees are of particular interest to production forestry and native plant restoration, especially where seedlings are planted on marginal or degraded forest sites and compete with aggressive non-native vegetation (Lawson and Michler, 2014). While mean annual precipitation can exceed 1,200 mm in the Puget lowlands and 2,500 mm in the Cascade foothills of western Washington, a pronounced summer drought is common (Waring and Franklin, 1979; Brooks et al., 2002). Summer droughts are expected to lengthen alongside temperature increases in the Pacific Northwest (PNW; Mote and Salathe, 2010), and Washington State’s 2015 drought resulted in plantation failure and economic losses in the agricultural sector (Cook et al., 2018). Extreme drought years similar to 2015 are projected to increase in frequency in the PNW (Marlier et al., 2017). Planted seedlings require reliable moisture during the growing season to facilitate root egress, to couple with site hydrology, and to enable long-term survival and growth (Grossnickle, 2012). Microbial endophytes influence seedling phenotypic plasticity, including drought resistance (Ryan et al., 2008). Developing drought-resistant planting materials would reduce plantation failure.

The application of endophyte consortia at a scale relevant to operational forestry practices, and for the purposes of improving seedling performance, is poorly studied as past research primarily focused on single-strain–single-host interactions (Chanway et al., 1991, 2000; Anand and Chanway, 2013; Anand et al., 2013). Introducing an endophyte consortium to developed planting stock resulted in enhanced root length, weight, and overall plant dry mass (Khan et al., 2015). However, previous research indicates a lag-time between inoculation and a detectable presence of endophytes (Parker and Dangerfield, 1975; Anand and Chanway, 2013), or a positive growth response (Khan et al., 2015) in tree species. Lag time could be reduced by inoculation at earlier plant developmental stages. Additions of inoculum to the rhizosphere or seeds have been shown to improve emergence in conifers (Chanway et al., 1991) and other plants (Muñoz-Rojas et al., 2018).

We used a field trial and subsequent greenhouse experiments to assess three objectives: (1) detect potential growth benefits or stress mitigation effects of microbial endophytes isolated from Salicaceae, Pinaceae, and Cupressaceae; (2) demonstrate the use of reproducible and reliable metrics to quantify the morpho-physiological expressions of these possible benefits; and (3) determine if the timing and means of application influences inoculation potential and growth/development of seedlings. Endophyte selection for the following experiments was based on previous studies demonstrating plant growth promoting (PGP) abilities under nitrogen and phosphorous limitation and drought stress on several crops (Khan et al., 2012, 2015, 2016; Knoth et al., 2013; Kandel et al., 2015; Rho et al., 2018a).

## Materials and Methods

### Plant Collection and Endophyte Strains

Four consortia were assembled from an assortment of 18 endophyte strains, and were categorized as either the “Poplar mix,” the “Willow mix,” or the “Conifer mix,” reflecting the source genus from which they were isolated (Table 1). Consortium 1 contained a poplar (*Populus* spp.) genus endophyte mix used in Experiment 1 (WP1, WP9, WP19, WPB, PTD1, PTD3). Consortium 2 contained a willow (*Salix* spp.) and poplar genus endophyte mix used in Experiment 1 (WP5, WW5, WW6, WW7). Consortium 3 contained a newly developed conifer genus endophyte mix used in Experiments 1 and 3 (TPSK3, TPSK5, TPSN7, PMPF3, PMSK6, PSSN1). Consortium 4 consisted of a combination of Consortium 1 and 2 with the addition of strains WP7 and PDN3, resulting in a broad mix of both poplar and willow genus endophtyes, and was used in Experiments 1 and 2.

**TABLE 1:** Microbial endophyte strains and consortia used across three experiments with conifers *Pseudotsuga menziesii* and *Thuja plicata.*

**Experiment applied**	**Components of consortium**	**Mix name**	**Strain name**	**Closest 16S rRNA match**	**References**
1 and 2	1 and 4	Poplar	WP1	*Rhodotorula graminis*	4, 6, 9, 10, 11, 12, 13, 14, 16
1 and 2	1 and 4	Poplar	WP9	*Paraburkholderia* sp.	3, 6, 10, 11, 14
1 and 2	1 and 4	Poplar	WP19	*Acinetobacter calcoaceticus*	3, 6, 10, 11, 12, 13, 14
1 and 2	1 and 4	Poplar	WPB	*Burkholderia vietnamiensis*	3, 6, 11, 12, 13, 14, 15
1 and 2	1 and 4	Poplar	PTD1	*Rhizobium tropici bv populus*	1, 6, 9, 10, 11, 12, 13, 15
1 and 2	1 and 4	Poplar	PTD3	*Rhodotorula mucilaginosa*	9, 13, 16
1 and 2	4	Poplar	PDN3	*Enterobacter* sp.	2, 8
1 and 2	4	Poplar	WP7	*Enterobacter* sp.	3, 13, 15
1 and 2	2 and 4	Willow	WP5	*Rahnella* sp.	3, 6, 7, 9, 11, 12, 13, 14
1 and 2	2 and 4	Willow	WW5	*Sphingomonas yanoikuyae*	6, 10, 11, 13, 14
1 and 2	2 and 4	Willow	WW6	*Pseudomonas putida*	6, 10, 11, 13, 14, 15
1 and 2	2 and 4	Willow	WW7	*Sphingomonas* sp.	6, 5, 10, 11, 12, 13
1 and 3	3	Conifer	TPSK3	*Paraburkholderia* sp.	This work
1 and 3	3	Conifer	TPSK5	*Herbaspirillum* sp.	This work
1 and 3	3	Conifer	TPSN7	*Burkholderia* sp.	This work
1 and 3	3	Conifer	PMPF3	*Rahnella* sp.	This work
1 and 3	3	Conifer	PMSK6	*Rahnella* sp.	This work
1 and 3	3	Conifer	PSSN1	*Paraburkholderia* sp.	This work

*Details of molecular characterization of the endophytes are found in the original research reports as noted 1, Doty et al. (2005); 2, Doty et al. (2017); 3, Doty et al. (2009); 4, Firrincieli et al. (2015); 5, Kandel et al. (2017a); 6, Kandel et al. (2015); 7, Kandel et al. (2017b); 8, Kang et al. (2012); 9, Khan et al. (2012); 10, Khan et al. (2015); 11, Khan et al. (2016); 12, Knoth et al. (2013); 13, Knoth et al. (2014); 14, Rho et al. (2018b); 15, Xin et al. (2009a); 16, Xin et al. (2009b).*

All strains from Consortium 1 and 2 were previously isolated and assessed for PGP traits and promoted growth and drought tolerance on a variety of crops (Khan et al., 2012, 2015, 2016; Knoth et al., 2013; Kandel et al., 2015, 2017a, b; Rho et al., 2018b). Strains from Consortium 3 were isolated from *P. menziesii*, *T. plicata*, and Sitka spruce (*Picea sitchensis*) trees growing in the riparian zones of Snoqualmie (47°31′14.30″ N, 121°46′28.32″ W) and Skykomish (47°46′48.3240″ N, 121°30′01.3320″ W) rivers in Washington State. Conifer branch cuttings were surface disinfected following standard protocols (Doty et al., 2016), and endophytes were isolated by enriching stem and leaf sections in 25 ml NL-CCM broth (Rennie, 1981) for a week, sub-culturing in NL-CCM for a second round of enrichment, and plating 0.1 ml on NL-CCM agar. Individual colonies were streak-purified on NL-CCM agar, dominant colony types were grown in NL-CCM broth and stored at -70°C in 33% sterile glycerol. The strains were assessed for PGP traits including production of indole acetic acid (IAA), ability to solubilize inorganic phosphate, and production of siderophores (Khan et al., 2015). The isolates were tested for the presence of the nitrogenase subunit gene, *nifH*, via colony PCR with the *nifH* Universal Primer set (Bürgmann et al., 2004). Following agarose gel electrophoresis, the expected 371 bp products were extracted using the QIAquick Gel Extraction Kit (Qiagen) and re-amplified using the same primer set or the nested b1 primer set (Bürgmann et al., 2004). Endophyte strains were identified through 16S rRNA PCR amplification using 8F and 1492R primers. The final PCR products of *nifH* and the 16S rRNA gene were prepared for sequencing using EXOSAP-IT (USB Corporation). The PCR products were sequenced through Sanger sequencing by Genewiz Inc. and the resulting sequences were analyzed using the Basic Local Alignment Search Tool (BLAST) of the National Center for Biotechnology Information (NCBI). The strains were also tested for nitrogenase activity by the acetylene reduction assay (ARA) as described by Kandel et al. (2017a).

Included in Consortium 3 was Strain PMPF3, a conifer endophyte, collected at the Center for Sustainable Forestry at Pack Forest (46.8432° N, 122.3176° W) in September 2011. *P. menziesii* branch samples were surface-disinfected, crushed in N-free Murashige and Skoog broth (NFMS; Caisson Laboratories, Inc.), and plated on NFMS agar. Morphologically distinct colonies were streak-purified, grown in NFMS broth, and cryogenically stored. Endophyte inoculum suspensions for all the experiments were prepared accordingly following protocols described in Khan et al. (2016).

### Experiment 1: Field Trials

At Pack Forest, a fenced field trial was established following clearing of vegetation and plowing, on a drought-prone soil – Barneston gravelly coarse sandy loam, ∼300 m elevation, 1,500 mm annual precipitation, and 7–10°C average temperature (Soil Survey Staff, 2004). Seedlings of *P. menziesii* and *T. plicata* were cultivated from seeds of the same provenance as the planting location, and grown by Silvaseed Company (Roy, WA, United States) to meet commercial specifications. Immediately prior to transplanting, seedlings were randomized and then the roots soaked in one of five solutions containing either endophyte strain WP1, Consortium 1+2, Consortium 3, Consortium 4, or a mock inoculation (media control).

Seedlings of *T. plicata* were planted in March 2013, and *P. menziesii* in March 2014. Each species was planted into a separate rectangular experimental layout (single block), consisting of evenly spaced rows (1-m between seedling) and columns (2-m between seedlings), and following a complete randomized design. For each species, each treatment consisted of 50 replicates (*n* = 50) comprising an overall block of 250 (*N* = 250) randomly distributed seedlings. After transplant, we used mechanical weeding to control competing invasive species. For *T. plicata*, seedling survival (%), height (cm), and root-collar diameter (RCD; mm) were assessed following one, two, and five growing seasons, or 180, 540, and 1,600+ days after inoculation (DAI), respectively. For *P. menziesii*, seedling survival (%), height (cm), and RCD (mm) were assessed following four growing seasons following 1,200+ DAI.

### Experiment 2: Seedling Tolerance to Varying Drought Intensities

*Pseudotsuga menziesii* and *T. plicata* bareroot seedlings (i.e., “plug+1” using forestry nomenclature relevant to culturing technique), derived from local provenance, were cultivated at Silvaseed Company and lifted from nursery beds 1 month prior to experimentation. Seedlings were sorted to eliminate outliers, then roots were rinsed free of growth medium using filtered greenhouse irrigation water immediately prior to transplant into 10.65 l pots (TP815R, Stuewe and Sons, Corvallis, OR, United States). During experimentation, seedlings were grown in sphagnum peat (Sunshine^®^ Mix #4), amended with a low-dose control release fertilizer (CRF; Osmocote^®^ 13-10-13, Scotts-Sierra Horticultural Products Co.).

For inoculation, each endophyte in Consortium 1 and 2 was grown in MG/L for 24 h and the cells harvested by centrifugation at 8,000 rpm at 4°C for 10 min and resuspended in half strength Hoagland solution (Khan et al., 2012). A 100 ml suspension containing a mix of the resuspended cells was poured into the seedling rhizosphere while the uninoculated control seedlings received 100 ml of sterile half-strength Hoagland solution.

**FIGURE 1:**
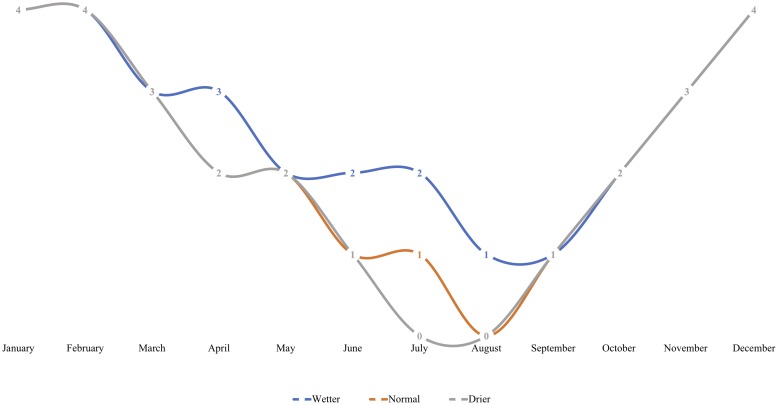
An irrigation scheme was developed to simulate regionally relevant *in situ* rhizosphere moisture conditions with varying degrees of “pronounced summer drought” intensity for *Pseudotsuga menziesii* and *T. plicata* seedlings in Experiment 2. Seedlings were assigned to either a “wetter,” “normal,” or “drier” irrigation treatment for the duration of the experiment. Irrigation was moderated using timing, where frequency of monthly saturation events corresponds to a respective treatment. Each number on a line indicates number of times a container was saturated to capacity per month (at regularly spaced intervals) for the duration of the 18-month trial that began in April of 2015.

Seedlings were subject to stable greenhouse conditions (Center for Urban Horticulture, Seattle, WA, United States) including artificially extended photoperiod of 18 h, stable 20/14°C day/night temperature, and controlled relative humidity (65–85%). Seedlings were arranged in two distinct blocks on a bench, each containing either control or inoculated treatment groups. Within each block, seedlings were subject to one of three nested irrigation treatments, and watered to saturation on a fixed schedule by treatment, corresponding to “wetter,” “drier,” or “normal” rhizosphere moisture conditions (Figure 1). The fixed-frequency irrigation scheme mimicked true field conditions of increasing summer drought in the PNW.

The experiment followed a factorial design with endophyte treatments (inoculated vs. control) contributing two levels and irrigation treatments contributing three levels; endophyte and irrigation factors were modeled as a categorical variable with six levels. Each species was cultured in a separate experiment, consisting of two adjacent greenhouse benches per species. Endophyte-inoculated seedlings were separated from uninoculated control seedlings, but grouped in randomized blocks according to irrigation treatment. Strict phytosanitary practices were implemented – including flushing water lines and bleach/alcohol surface-disinfection of instruments between endophyte treatment groups – during irrigation and handling of the plants for measurements. Each nested irrigation treatment consisted of 20 seedling replicates (*n* = 20 for endophyte × irrigation treatment), amounting to 120 potted seedlings per species (*N* = 120). The two species’ experiments were arranged identically, and to reduce a potential “edge-effect,” seedlings were re-randomized within each block prior to each irrigation event.

Seedling survival, height, and RCD were assessed following transplant, again after the first growing season (180 DAI) and following the second growing seasons (540 DAI). A destructive harvest ended the experiment (at 540 DAI). At that time, seedlings’ roots were rinsed free from the growing medium and oven dried for 72 h at 60°C for subsequent measurements of root and shoot dry mass (SDM) (g).

Plant physiological status (i.e., stress response) was estimated through measurements of chlorophyll fluorescence and relative chlorophyll content: once following transplant, once 180 DAI (mid-winter), and monthly 360–450 DAI (second growing season). Fluorescence was measured with a portable fluorometer (OS30P+, Opti-Sciences, Inc., Hudson, NH, United States). First, foliage was dark adapted for 20+ min, then a weak modulating light was used to calculate *F*_o_ (minimum fluorescence), followed by an intense saturating light used to calculate *F*_m_ (maximum fluorescence). Variable fluorescence (*F*_v_ = *F*_m_−*F*_o_) was calculated by the instrument from the chlorophyll fluorescence (*F*_v_/*F*_m_) ratio which reflects the potential quantum efficiency of PSII – an indicator of plant photosynthetic performance and a measure of photo-inhibition when seedlings are stressed (Maxwell and Johnson, 2000).

Relative chlorophyll content was measured in SPAD units using a Konica Minolta SPAD 502 meter (Konica Minolta, Ramsey, NJ, United States). SPAD measurements indicate relative chlorophyll content of leaf tissue by measuring transmittance of the tissues in the red and infrared regions (Bussotti and Pollastrini, 2015). The resulting arbitrary SPAD meter units are a suitable proxy for chlorophyll content (Uchino et al., 2013).

Whole plant surveys from three points per plant resulted in whole plant *F*_v_/*F*_m_ and SPAD values. *P. menziesii* measurements were derived from new needles which had matured post-transplant and post-bud break. For *T. plicata*, the latest developed sprays were selected for measurement.

### Experiment 3: Seedling Growth and Development in the Presence or Absence of Fertilizers

*Pseudotsuga menziesii* and *T. plicata* seed collected from Pack Forest and stored at Silvaseed Company were stratified for 28 days at 1–2°C. Stratified seeds were soaked for 24 h in aerated water and then immersed in the inoculum suspension or sterile N-free medium (control), for 6 h prior to sowing and placed on a shake table. Seeds were subsequently strained and placed on a Petri dish.

Seeds were sown into 656 cm^3^ D40 Deepots, filled with sphagnum media (Sunshine^®^ Mix #4), and top-dressed with perlite. Half of the containers were amended with CRF (Osmocote^®^ 13-10-13) while the remaining half received no amendment. Mist irrigation was applied twice daily until emergence. For the remainder of the experiment, sub-irrigation using flowtrays was applied to each treatment block of seedlings within a group. This irrigation method minimized risk of cross-contaminating control seedlings and minimized loss of control-release fertilizer from leaching. A pure water flush was employed as needed to maintain slightly acidic media conditions near a pH of 6. Moisture was maintained at ∼75% or greater field capacity for the duration of the spring and summer. Container medium was maintained at ∼60% field capacity in the autumn and winter months.

Seedlings were arranged on two distinct greenhouse tables, each containing even replicates of (4 × 5) sortable trays (i.e., blocks) of either “un-inoculated control” or “inoculated” treatment groups. Each tray was assigned one of two nested fertilizer treatments, “fertilized” or “unfertilized (control).” Seedlings were subject to the same stable greenhouse conditions described for Experiment 2.

The design was factorial, with an endophyte group (Consortium 3) and two fertilizer treatments, thus endophyte and fertilizer factors were modeled as categorical variables with four levels. Each species was cultured in a separate experiment, consisting of two adjacent greenhouse benches per species. Endophyte-inoculated seedlings were separated from uninoculated control seedlings, but grouped in randomized blocks (trays) according to fertilizer treatment. Strict phytosanitary practices were implemented as described for Experiment 2. Each species was treated as a separate experiment, comprised of 60 seedling replicates (20/tray) per treatment combination (*n* = 60 for endophyte × fertilizer treatment), for a total of 240 container grown seedlings per species (*N* = 240). Seedlings were maintained in a fixed arrangement within each tray; however, trays were randomized on each greenhouse bench following each irrigation event to minimize “edge-effect.”

Germination rate was monitored between treatment groups. Then, monthly measures 30–120 DAI and 360–450 DAI of seedling height, RCD, *F*_v_/*F*_m_, and SPAD of each growing season followed protocols described above. A destructive harvest was conducted 1 month after germination (50 DAI), and again at the end of the experiment (540 DAI). First-order lateral roots (FOLRs; Davis and Jacobs, 2005) were counted on each month-old seedling. At the final harvest FOLR and first-order branches (those diverging from the main stem; FOB) of each seedling were sampled prior to oven drying tissue for dry mass measurements.

### Statistical Analysis

For the field trials comprising Experiment 1, response variables were analyzed with a one-way analysis of variance (ANOVA). For the greenhouse work in Experiments 2 and 3, response variables for each species were examined separately with a two-way ANOVA. Pair-wise comparisons of means were made using Tukey’s honestly significant differences (HSD) test (α = 0.05) between inoculated and mock-inoculated seedlings at each treatment level using R statistical system (R Core Team, 2015) with the “lme4” and “lsmeans” packages.

## Results

### Conifer Endophyte Characterizations

Six additional endophyte strains from conifers were selected for inclusion in the study. *Paraburkholderia* sp. strain TPSK3 was isolated from *T. plicata* near the Skykomish River. It produced the auxin, IAA, solubilized phosphate, produced siderophores, and was positive for presence of the *nifH* nitrogenase subunit. The strain reduced acetylene to ethylene, indicative of N-fixation, to a level of 12 nmol ethylene, about 2% the level of the positive control strain, *Azotobacter vinelandii*. *Herbaspirillum* sp. strain TPSK5, also isolated from the same source, produced IAA, solubilized phosphate, was positive for *nifH* (matching that of *Herbaspirillum* sp. With an *E*-value of 1*e*−155) but negative in the conditions of the ARA. *Burkholderia* sp. strain TPSN7, isolated from *T. plicata* near the Snoqualmie River, produced IAA and was positive for the presence of the nifH gene, matching that of *Burkholderia xenovorans* with an *E*-value of 1e−31. It was also positive in the ARA (18 nmol ethylene, 3% the level of the positive control). *Rahnella* sp. strain PMPF3, isolated from Douglas-fir from Pack Forest, solubilized phosphate and was positive for *nifH* (matching that of *B. xenovorans* with an *E*-value of 0.0) but negative in the conditions of the ARA. *Burkholderia* sp. strain PMSK6, isolated from Douglas-fir at the Skykomish River, produced IAA and was also positive for *nifH* but negative in the ARA. *Paraburkholderia* sp. strain PSSN-1, isolated from Sitka spruce at the Snoqualmie River, was especially active in phosphate solubilization but negative for ARA and *nifH* PCR but did produce IAA.

### Experiment 1: Field Trials

All *T. plicata* seedlings planted in the spring of 2013 survived to measurement in the fall (180 DAI). Mean height was lower for seedlings from the single-strain WP1 treatment than those from Consortium 1+2, 3, and 4, however, neither treatment was significantly different in height from control seedlings (Table 2). *T. plicata* RCD was similar after the first growing season. Following a second growing season (540 DAI), seedlings treated with the Consortium 3 had a higher survival rate than those treated with the WP1 or Consortium 1+2 and 4; however, the survival rate of *T. plicata* seedlings receiving the endophyte treatments was similar to the control group. In 2017, following five growing seasons, the survival rate among endophyte-treated seedlings was statistically similar to the control group, despite the trend showing Consortium 3 seedlings with a slightly higher survival rate. RCD growth of *T. plicata* treated with the Consortium 4 was significantly greater than the control treatment and Consortium 3 at 1,600+ DAI.

**TABLE 2:** Summary of field trials at Pack Forest, where *T. plicata* and *P. menziesii* seedlings were inoculated with one of four endophyte consortia prior to transplant, then monitored intermittently from 2013/14 to 2017, respectively.

		**Treatments**	**WP1 only**		**Consortium 1+2**		**Consortium 3**		**Consortium 4**		**Control**	
*Thuja plicata*	2013 (180 DAI)	Height growth (cm)	39.84	(1.58)a	43.26	(1.62)ab	46.18	(1.52)b	43.61	(1.56)ab	41.55	(1.56)ab
		RCD growth (mm)	5.31	(0.51)a	5.14	(0.52)a	5.46	(0.49)a	5.04	(0.50)a	6.02	(0.50)a
	2014 (540 DAI)	Height growth (cm)	39.92	(1.68)a	45.53	(1.68)a	43.43	(1.59)a	41.65	(1.63)a	43.21	(1.63)a
		RCD growth (mm)	5.1	(0.51)a	5.36	(0.51)a	6.19	(0.49)a	5.09	(0.50)a	5.2	(0.50)a
		Survival (%)	74a		74a		82b		78ab		78ab	
	2017 (1,600^+^DAI)	Height growth (cm)	80.6	(6.53)a	74.2	(6.53)a	78.11	(6.05)a	86.59	(7.15)a	76.45	(6.53)a
		RCD growth (mm)	13.63	(0.99)ab	12.57	(0.99)ab	12.16	(0.92)a	16.17	(1.08)b	11.99	(0.99)a
		Survival (%)	24ab		24ab		30b		20a		24ab	
*Pseudotsuga menziesii*	2017 (1,200^+^DAI)	Height growth (cm)	43.9	(17.16)a	49.98	(7.67)a	56.1	(17.16)a	72.95	(12.13)a	^*^	
		RCD growth (mm)	10	(3.34)a	11.22	(1.49)a	13.9	(3.34)a	15.75	(2.36)a		
		Survival (%)	2a		10b		4a		4a			

*Data were collected following the growing season in the autumn, list as days after inoculation (DAI). Unweighted mean values of delta growth and root-collar diameter (RCD) variables and percent survival rates (SE). The same adjacent letter indicates that means are not different (alpha = 0.05) among the column for each species. No data were collected in the interim years for *P. menziesii* from planting through 2017; as the experiment was initially abandoned after severe site conditions and other logistical constraints.*

*Pseudotsuga menziesii* seedlings were chlorotic 6 weeks after transplant, suggesting a negative effect of the site, with the symptoms matching that of infection with *Phellinus sulphurascens*, a fungal pathogen. The poor condition of seedlings led to abandonment until a follow-up visit in 2017 (1,200+ DAI), which yielded few survivors, but all survivors were derived from endophyte treatment groups. The highest survival rate was 10%, from the group of *P. menziesii* seedlings treated with Consortium 1+2. Survivors from all treatment groups were not statistically different in height or RCD (Table 2).

### Experiment 2: Seedling Tolerance to Varying Drought Intensities

#### Survival

There were no statistically significant differences in survival rate between species and treatment groups after the first growing season, although an overall trend in the data shows a higher rate of survival of endophyte-inoculated seedlings at the end of that first second growing season. *P. menziesii* seedling survival was not statistically different between treatment and control groups after the first growing season. However, after two seasons, inoculated *P. menziesii* in the normal (i.e., typical seasonal drought) treatment group had significantly better (*p* ≤ 0.05) survival rates than the uninoculated control group (Tables 3, 4). An interaction effect (*p* = 0.03) was detected for *T. plicata* seedlings in survival following the first and second season across treatment groups. Inoculated *T. plicata* seedlings had a higher survival rate following two growing seasons (*p* ≤ 0.001), and water availability modulated that survival rate in both years. Specifically, seedlings survived at a higher rate (*p* ≤ 0.001) in the normal and drier moisture regime in the inoculated group, with 50% and 80% improvements, respectively. *T. plicata* survival was 100% in wet treatments both with and without endophytes.

**TABLE 3:** Summary of *P. menziesii* and *T. plicata* seedling mortality, growth, and physiological metrics in response to drought treatments and controls at 6 months after transplant (180 DAI).

**Species**	**Exp. Treatments**		**Survival (%)**	**Fv/Fm**		**SPAD**		**Height growth (cm)**		**RCD growth (mm)**	
*Pseudotsuga menziesii*	Control	Wetter	90a	0.811	(0.010)a	25.7	(5.9)a	18.95	(2.22)a	5.49	(0.66)b
		Normal	50a	0.808	(0.017)a	23.5	(5.40)a	16.75	(2.22)a	1.25	(0.66)a
		Drier	80a	0.813	(0.020)a	20.6	(5.31)a	15.75	(2.22)a	−0.30	(0.66)a
	Inoculated	Wetter	80a	0.818	(0.021)a	28.1	(5.40)a	15.10	(2.22)a	4.58	(0.66)b
		Normal	60a	0.817	(0.014)a	23.2	(5.40)a	17.15	(2.22)a	−1.43	(0.66)a
		Drier	100a	0.815	(0.022)a	23.7	(5.9)a	12.20	(2.22)a	−0.51	(0.66)a
*Thuja plicata*	Control	Wetter	100a	0.816	(0.033)a	34.3	(3.56)a	20.20	(1.09)b	3.32	(0.63)bc
		Normal	50b	0.803	(0.029)a	29.1	(3.30)a	11.60	(1.09)a	1.62	(0.63)b
		Drier	60b	0.814	(0.026)a	30.1	(3.30)a	9.70	(1.09)a	0.90	(0.63)b
	Inoculated	Wetter	100a	0.821	(0.017)a	32.9	(3.45)a	18.15	(1.09)b	4.53	(0.63)c
		Normal	80a	0.800	(0.021)a	34.5	(3.45)a	13.10	(1.09)a	0.79	(0.63)b
		Drier	90a	0.801	(0.022)a	29.7	(3.45)a	11.60	(1.09)a	−1.90	(0.63)a

*Values from Fv/Fm and SPAD represent a final seasonal measurement event, following the full duration of “precipitation” treatments, and means are based on an average (*n* = 4/plant) of sun and shade leaves. Percent’s and unweighted mean values of delta growth variables (SE). The same adjacent letter indicates that means are not different (alpha = 0.05) among the column for each species.*

**TABLE 4:** Summary of *P. menziesii* and *T. plicata* seedling mortality, growth, and dry tissue metrics in response to drought treatments and controls following 18 months experimental duration (540 DAI).

**Species**	**Exp. Treatments**		**Survival (%)**	**Height growth (cm)**		**RCD growth (mm)**		**Root dry mass (g)**		**Shoot dry mass (g)**	
*Pseudotsuga menziesii*	Control	Wetter	90a	28.44	(2.88)b	6.30	(0.84)c	44.67	(6.31)ab	92.27667	(8.95)b
		Normal	20b	24.00	(3.86)ab	4.99	(1.06)bc	24.0733	(6.31)a	70.40333	(8.95)ab
		Drier	70a	18.93	(3.26)ab	0.40	(0.90)a	24.3633	(6.31)ab	49.3633	(8.95)a
	Inoculated	Wetter	80a	18.19	(3.05)ab	6.07	(0.80)c	60.75	(6.31)b	97.16667	(8.95)b
		Normal	60a	16.08	(3.52)ab	1.61	(0.97)ab	36.12	(6.31)ab	64.87	(8.95)ab
		Drier	100a	15.75	(2.73)a	0.19	(0.75)a	31.55	(6.31)ab	74.81	(8.95)ab
*Thuja plicata*	Control	Wetter	100a	44.60	(4.003)ab	10.49	(1.03)b	129.770	(10.65)bc	99.907	(10.73)ab
		Normal	30b	29.24	(4.785)a	6.67	(1.23)ab	81.080	(13.05)ab	61.555	(13.14)a
		Drier	0b	46.50	(4.22)ab	7.29	(1.08)ab	^*^		^*^	
	Inoculated	Wetter	100a	44.10	(4.003)ab	10.96	(1.03)b	164.250	(10.65)c	133.953	(10.73)b
		Normal	80a	56.63	(4.476)b	7.62	(1.15)ab	56.990	(10.65)a	102.033	(10.73)ab
		Drier	90a	47.28	(4.22)ab	4.66	(1.08)a	44.920	(10.65)a	83.410	(10.73)ab

Percent and unweighted means of delta growth variables (SE). The same adjacent letter indicates that means are not different (alpha = 0.05) among the column for each species. ^*^Complete mortality early enough in experimentation invalidated dry tissue mass measurement of samples subject to this treatment combination.

#### Growth

Height growth at ∼180 DAI was similar between inoculated and uninoculated seedlings for both species. *P. menziesii* seedlings had negative mean RCD growth (−1.43 mm) in the normal inoculated treatment compared to positive mean growth (1.25 mm) in the un-inoculated control group (Table 3). *T. plicata* seedlings had lower (*p* ≤ 0.05), and negative mean RCD growth (−1.90 mm) in the drier treatment when inoculated, compared to positive mean growth (0.90 mm) in the reciprocal control group. Mean growth was higher among wetter moisture regime treatments compared to lower moisture treatments for *T. plicata* (*p* ≤ 0.05).

Moisture treatment effects continued in the second growing season, as growth increased with water availability. For *P. menziesii*, increasing moisture increased RCD, RDM, and SDM (*p* ≤ 0.001). Endophyte treatment reduced height growth in the second growing season (Table 4). No interaction effect was detected between the endophyte treatment for height, RCD, root dry mass (RDM), or SDM for *P. menziesii* seedlings at 540 DAI.

Positive growth trends among *T. plicata* seedling’ RCD, RDM, and SDM in 2016 appear to be modulated by moisture as well; with decreased drought stress resulting in higher mean growth among inoculated seedlings (Table 4). Significant interaction effects were detected between the main effects of height (*p* = 0.03) and RDM (*p* = 0.003) response variables. Height growth was significantly greater (*p* = 0.005) among the normal moisture regime seedlings in the inoculated group (56.63 cm) versus the corresponding un-inoculated treatment group (29.24 cm).

#### Physiology

Seedlings showed limited stress in the first growing season with *F*_v_/*F*_m_ ratios and SPAD values within an expected “healthy” range and with no differences among treatment groups (Table 3). Table 3 reports final mean *F*_v_*/F*_m_ and SPAD values at 180 DAI, which remained steady for the duration of the growing season.

**FIGURE 2:**
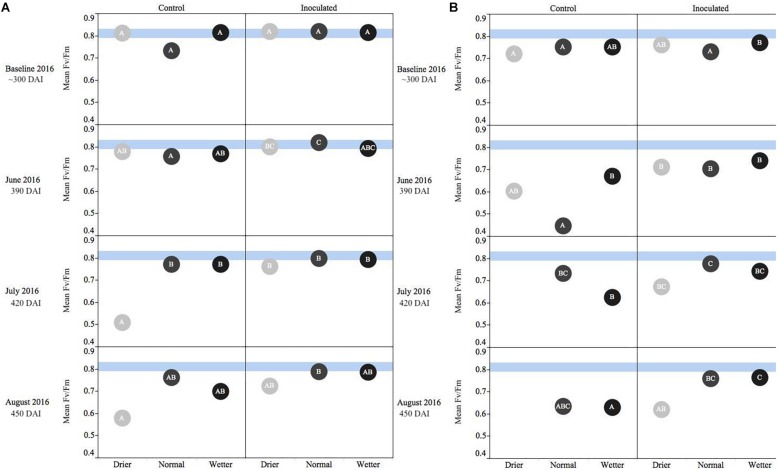
Seasonal progression of mean values representing chlorophyll fluorescence ratios (Fv/Fm) for *P. menziesii* **(A)** and *T. plicata* **(B)** seedlings, measured the second growing season after transplant, approximately 390–450 DAI. Baseline measurements were derived from combining mid-winter/early-spring values, and subsequent monthly measurements capture onset and progression of seasonal drought simulation at different intensities from June to August 2016. Light blue band represents healthy performance range for this species (0.790–0.830), and gradient of light gray to black corresponds to moisture regime assigned to drier, normal, and wetter, respectively. The same embedded letter indicates that means are not different (alpha = 0.05) across a pane representing the measurement period.

For both species, measurements at the start of the second growing season (360 DAI) were used as baseline readings. The *F*_v_/*F*_m_ mean values for *P. menziesii* were not different at baseline. In June (390 DAI), both moisture availability and inoculation are significant (*p* ≤ 0.001) drivers of fluorescence, and increased water availability corresponds to values persisting in a stable range corresponding to the baseline. In July and August (420–450 DAI), a significant (*p* = 0.0001) interaction effect is detected and both moisture and inoculation remain significant (*p* ≤ 0.01) drivers of *F*_v_*/F*_m_ values. In June (390 DAI), the inoculated seedlings subject to the normal moisture regime were performing significantly (*p* ≤ 0.05) better (maintaining “normal” range) than seedlings in the corresponding un-inoculated control group (Figure 2A). In July – at the onset of peak moisture stress (Figure 1) – the inoculated seedlings in the drier treatment maintained a higher *F*_v_*/F*_m_ ratio than those in the reciprocal un-inoculated control group. In August, the trend of higher *F*_v_*/F*_m_ ratio among inoculated seedlings persisted and was pronounced when compared to the driest moisture regimes; however, the outcomes were not statistically significant.

**FIGURE 3:**
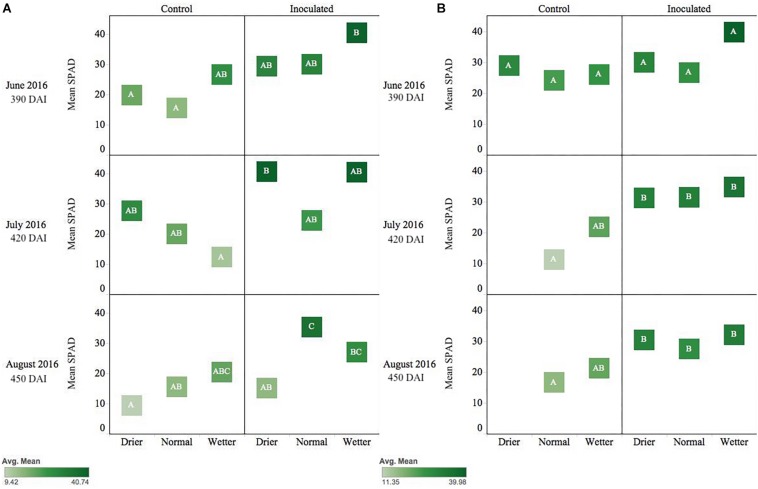
Seasonal progression of mean relative leaf chlorophyll content (SPAD) values for *P. menziesii* **(A)** and *T. plicata* **(B)** seedlings, measured the second growing season after transplant, approximately 390–450 DAI. Monthly measurements capture onset and progression of seasonal drought simulation at different intensities from June to August 2016. The same embedded letter indicates that means are not different (alpha = 0.05) across a pane representing the measurement period.

*Pseudotsuga menziesii* seedlings were consistently greener and had higher SPAD values among inoculated seedlings compared to controls when measured in June, July, and August (390–450 DAI). The endophyte treatment main effect was statistically significant (*p* ≤ 0.01) for the duration of those months, and water was significant in June (*p* = 0.05) and August (*p* = 0.003). In August, the inoculated seedlings in the normal moisture regime had significantly (*p* ≤ 0.01) higher SPAD readings than seedlings in the un-inoculated reciprocal moisture regime (Figure 3A).

An interaction effect was detected for the mean values of *T. plicata F*_v_/*F*_m_ ratios at approximately 300 DAI, when baseline (outside of growing season) readings were collected. However, no significant differences or biologically meaningful trends were apparent at the onset of the second growing season. The main effects were both significant (*p* ≤ 0.05) drivers of differences for June through August (390–450 DAI), and a significant (*p* ≤ 0.001) interaction effect was apparent in July. A trend of higher *F*_v_/*F*_m_ ratios persisted among inoculated seedlings compared to the control group in July and August. Further, significant (*p* ≤ 0.05) differences were detected between the normal and drier moisture regime treatment groups in July and this difference became significant (*p* ≤ 0.05) between the wetter moisture regime group in August (Figure 2B).

Inoculated *T. plicata* seedlings were visibly greener, and had higher SPAD readings, than the control group from June to August (Figure 3B; 390–450 DAI). The endophyte treatment main effect had a significant effect on seedling greenness in July (*p* = 0.0005) and August (*p* = 0.003). Specifically, inoculated seedlings in the normal moisture regime had a significant (*p* ≤ 0.05) mean difference in SPAD units of 20.3 in July and 10.8 in August (Figure 3B) compared to the uninoculated control seedlings in the same regime, respectively. No interaction effect was detected for these months.

### Experiment 3: Seedling Growth and Development in the Presence or Absence of Fertilizers

#### Growth and Development

Germination was not significantly (*p* ≤ 0.05) different between conifer endophyte-inoculated and control groups of either species. At 50 DAI, inoculated *P. menziesii* seedlings had longer root radicles compared to the unfertilized control seedlings both with and without fertilization (*p* = 0.0001; Table 5). *T. plicata* seedling radicle lengths were similar among treatment groups. FOLR quantities were indistinguishable among treatments for either species at 50 DAI.

**TABLE 5:** Summary of radical length and first-order lateral root (FOLR) quantities by treatment combination for *P. menziesii* and *T. plicata* seedlings just over 1 month after sowing, June 2015 (∼50 DAI).

**Host species**	**Exp. Treatments**		**Radicle**		**FOLR (Qty)**	
			**length (mm)**			
*Pseudotsuga menziesii*	Control	Fertilized	74.073	(6.34)ab	5.0	(1.5)a
		Unfertilized	60.771	(6.34)a	1.0	(1.5)a
	Inoculated	Fertilized	92.749	(6.34)b	4.0	(1.5)a
		Unfertilized	92.276	(6.34)b	5.0	(1.5)a
*Thuja plicata*	Control	Fertilized	25.21	(2.04)a	2.0	(1.5)a
		Unfertilized	30.544	(2.04)a	4.0	(1.5)a
	Inoculated	Fertilized	31.57	(2.04)a	4.0	(1.5)a
		Unfertilized	29.61	(2.04)a	3.0	(1.5)a

*Seedlings were destructively sampled *n* = 10 per treatment combination. Mean values of both variables (SE) shown, and the same adjacent letter indicates that means are not different (alpha = 0.05) among the column for each species.*

Fertilizer treated seedlings were larger and greener than the unfertilized group, but there were no differences in morphological or physiological status between inoculated and un-inoculated treatment groups 360 DAI. *P. menziesii and T. plicata* both showed differences in growth attributes with most differences strictly attributable to fertilization effects at 390–450 DAI.

Interaction terms were not significant for the endophyte × fertilizer treatment effects for either species. However, growth among the inoculated seedlings was greater than the control for some measurements (Table 6).

**TABLE 6:** Summary of morphological variables by treatment combination for 18-month-old *P. menziesii* and *T. plicata* seedlings after final destructive harvest, October 2016 (540 DAI).

**Host species**	**Exp. Treatments**		**Height (cm)**		**RCD (mm)**		**FOLR (Qty)**		**FOB (Qty)**		**Root dry mass (g)**		**Shoot dry mass (g)**	
*Pseudotsuga menziesii*	Control	Fertilized	25.6	(0.80)b	6.03	(0.16)b	29.5	(2.9)ab	16.9	(1.0)b	9.83	(0.37)b	13.90	(0.50)b
		Unfertilized	7.54	(0.81)a	1.53	(0.17)a	26.0	(2.9)a	3.8	(1.0)a	7.33	(0.42)a	7.34	(0.55)a
	Inoculated	Fertilized	27.92	(0.80)b	6.33	(0.16)b	34.1	(2.9)ab	21.2	(1.0)c	11.74	(0.38)c	14.44	(0.50)b
		Unfertilized	8.84	(0.80)a	1.64	(0.16)a	38.6	(2.9)b	4.4	(1.0)a	7.75	(0.42)a	7.46	(0.55)a
*Thuja plicata*	Control	Fertilized	27.52	(1.24)b	5.14	(0.25)b	22.6	(1.9)a	14.1	(0.7)b	15.68	(0.63)b	17.23	(0.63)b
		Unfertilized	8.82	(1.22)a	1.32	(0.24)a	20.7	(1.9)a	4.1	(0.7)a	7.40	(0.52)a	7.25	(0.52)a
	Inoculated	Fertilized	34.45	(1.24)c	5.99	(0.24)b	30.1	(1.9)b	16.3	(0.7)b	17.08	(0.52)b	18.76	(0.52)b
		Unfertilized	8.43	(1.22)a	1.39	(0.24)a	26.7	(1.9)ab	5.3	(0.7)a	7.57	(0.52)a	7.36	(0.52)a

*Seedlings were destructively sampled *n* = 20 per treatment combination. Mean values of height, root-collar diameter (RCD), FOLRs, first-order branches (FOB), root dry mass (RDM), and shoot dry mass (SDM) variables (SE) shown. The same adjacent letter indicates that means are not different (alpha = 0.05) among the column for each species.*

At 540 DAI, fertilized, *P. menziesii* HT, RCD, RDM, SDM, and FOB growth was significantly (*p* ≤ 0.01) greater than unfertilized control seedlings in both endophyte treatment groups. The exception was the quantity of FOLR, which exceeded all other treatment groups (Table 6). Fertilized conifer-endophyte inoculated *P. menziesii* seedlings had larger RDM (*p* ≤ 0.01) and more FOB (*p* = 0.02) compared to the fertilized un-inoculated control group. Seedlings also had more FOLR in the inoculated unfertilized group compared to the control (*p* ≤ 0.01).

At 540 DAI, *T. plicata* seedlings in the fertilized treatments had grown more than unfertilized seedlings in the inoculated and control groups for each morphological response variable (*p* ≤ 0.05) except FOLR. Inoculated seedlings had more FOLR (*p* ≤ 0.01) than control seedlings in the presence and absence of fertilizer. Inoculated and fertilized *T. plicata* seedlings were (*p* ≤ 0.05) taller than seedlings in all other treatment groups. Similarly, the fertilized and inoculated group had more FOLR (*p* ≤ 0.05) than un-inoculated seedlings (Table 6).

**FIGURE 4:**
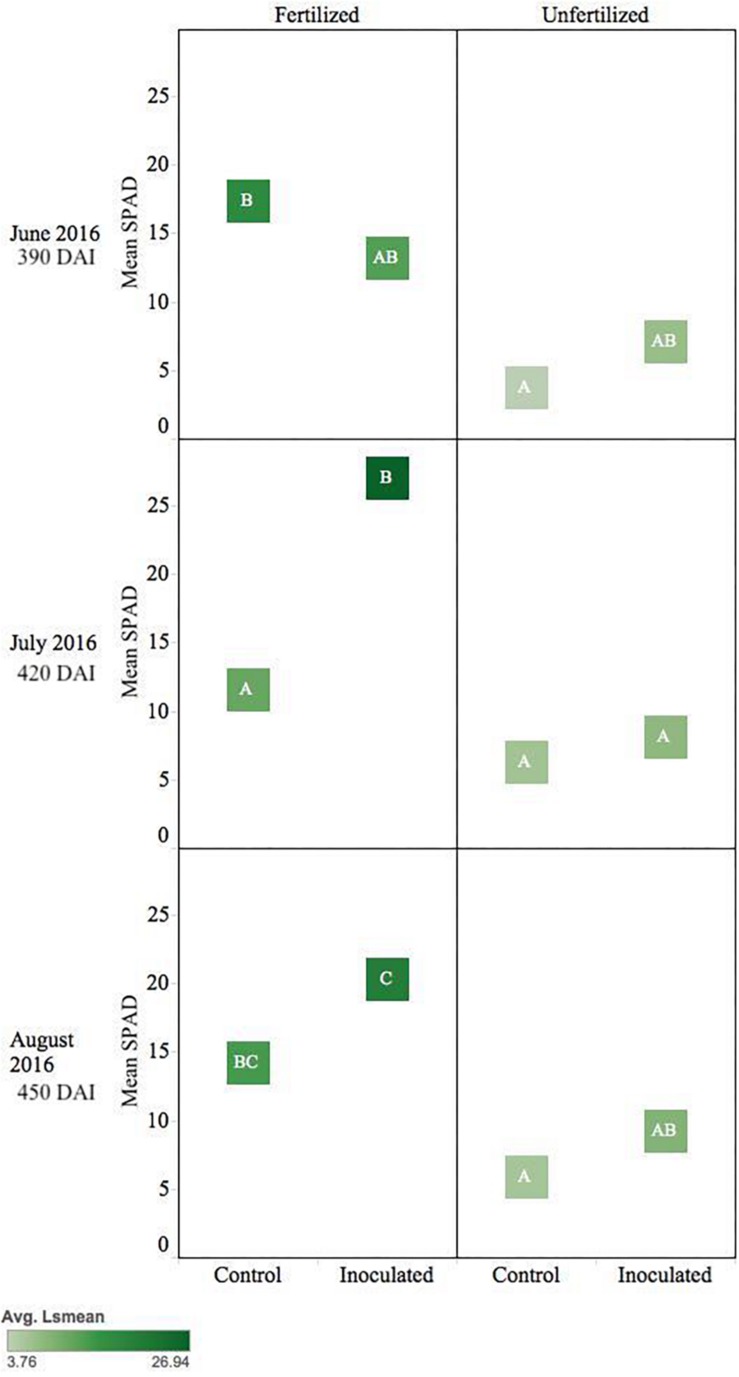
Seasonal progression of mean relative leaf chlorophyll content (SPAD) values for *P. menziesii* seedlings, measured the second growing season following germination, approximately 390–450 DAI. Monthly measurements capture increasing nutrient deficit in both fertilized and unfertilized treatment groups. The same embedded letter indicates that means are not different (alpha = 0.05) across a pane representing the measurement period.

*Pseudotsuga menziesii* seedlings showed no differences in *F*_v_/*F*_m_ among treatment groups >360 DAI. However, after 360 DAI, seedlings were visibly greener, and mean SPAD values began to separate between treatment groups. The fertilizer treatment effect continued in June, July, and August (390–450 DAI; *p* ≤ 0.01), and an interaction effect between fertilization and inoculation was detected for July (420 DAI, *p* = 0.02). The conifer endophyte-treated plants were greener in July and August (*p* < 0.05), the warmest months of experimentation. By August there were significant differences among groups ranked in order from highest to lowest: (1) inoculated fertilized, (2) control fertilized, (3) inoculated unfertilized, and (4) control unfertilized (*p* ≤ 0.05) (Figure 4).

**FIGURE 5:**
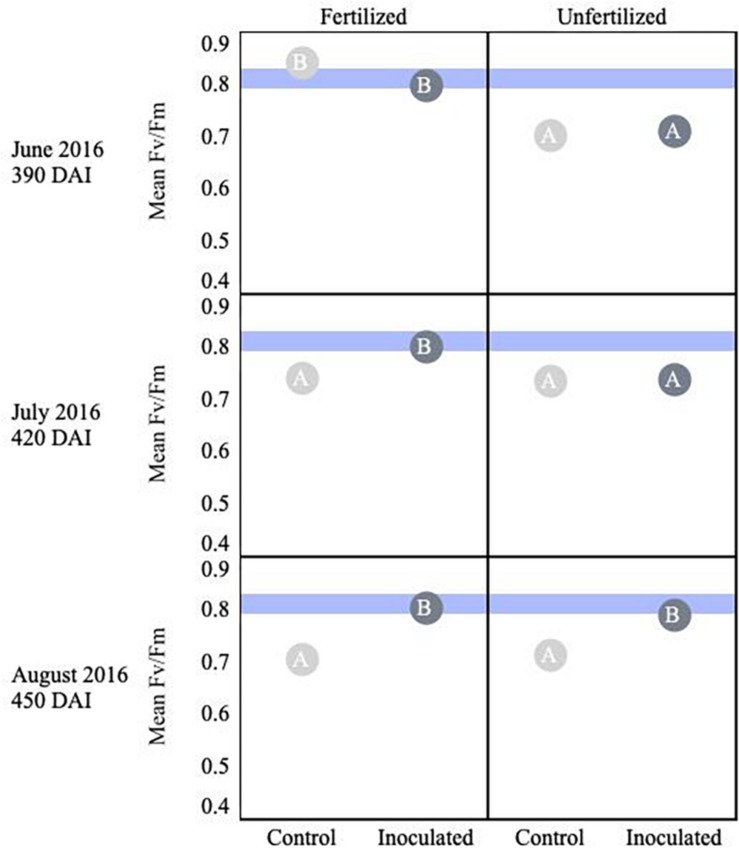
Seasonal progression of mean values representing chlorophyll fluorescence ratios (Fv/Fm) for *T. plicata* seedlings, measured the second growing season following germination, approximately 390–450 DAI. Monthly measurements capture increasing nutrient deficit in both fertilized and unfertilized treatment groups. Light blue band represents healthy performance range for this species (0.790–0.830), and gradient of light gray to black corresponds to moisture regime assigned to drier, normal, and wetter, respectively. The same embedded letter indicates that means are not different (alpha = 0.05) across a pane representing the measurement period.

*Thuja plicata* showed a treatment effect on overall plant stress (i.e., *F*_v_/*F*_m_) at 180 DAI. Early in the growing season fertilized plants were less stressed, but by August this reversed and inoculated seedlings were less stressed. Mean baseline values of fertilized seedlings appeared less stressed than unfertilized seedlings (*p* < 0.05). Approaching 360 DAI and persisting through the second growing season, inoculated seedlings showed lower stress (i.e., higher *F*_v_/*F*_m_ values, *p* ≤ 0.05) compared to control groups (Figure 5). An interaction effect was detected for July (*p* = 0.00036), as were main effects (*p* < 0.001). By 450 DAI, inoculated seedlings were less stressed than the reciprocal uninoculated control group (*p* ≤ 0.0001).

**FIGURE 6:**
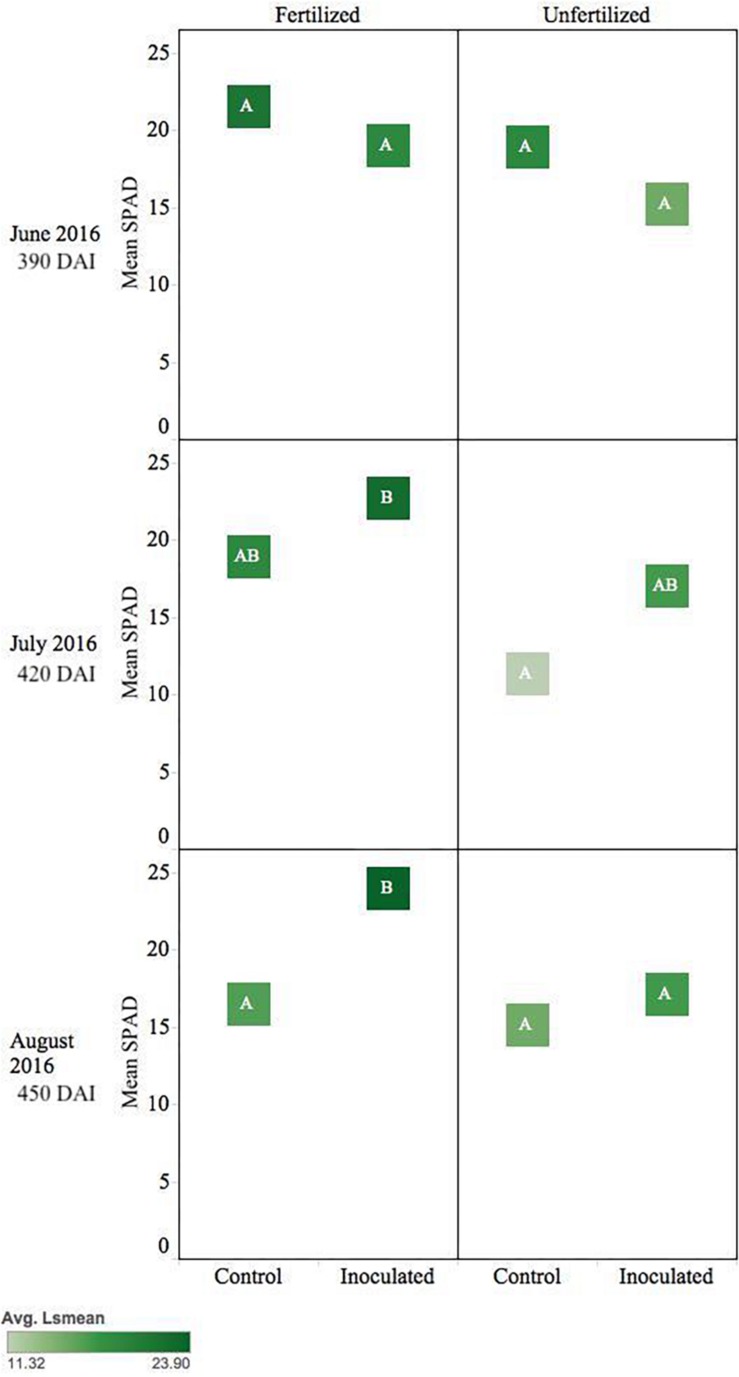
Seasonal progression of mean relative leaf chlorophyll content (SPAD) values for *T. plicata* seedlings, measured the second growing season following germination, approximately 390–450 DAI. Monthly measurements capture increasing nutrient deficit in both fertilized and unfertilized treatment groups. The same embedded letter indicates that means are not different (alpha = 0.05) across a pane representing the measurement period.

Inoculated unfertilized *T. plicata* seedlings were similar in greenness to the fertilized control ∼420 DAI. Unfertilized control seedlings were less green (*p* ≤ 0.05), while the fertilized inoculated seedlings were the greenest (i.e., higher SPAD). By August inoculated and fertilized seedlings were greener (*p* ≤ 0.05) than all other treatments (Figure 6).

## Discussion

We summarize three different experiments conducted over a 5-year period examining the effects of endophytes on *P. menziesii* and *T. plicata* seedling production. We hypothesized *a priori* that endophytic consortia would elicit a net positive survival, growth, and physiological response in host conifers when compared to uninoculated controls. Improvements in methodology developed over the 5-year period include: (1) changes in endophyte consortia used, (2) changes in conifer stock (seedlings and then seeds), and (3) significant increases in replication within treatment groups were adapted or built upon previous studies (Khan et al., 2015, 2016). The experiments, together, represent a reductive analysis focusing on limiting factors in seedling establishment and the role of endophytes in reducing those factors.

We observed endophyte effects when inoculating first year plug seedlings prior to planting and when growing plants after seed exposure prior to sowing. Inoculation of seed prior to planting is common in agricultural models, but less so in conifers (beginning with Chanway et al., 1991). Our study suggests inoculation in an endophyte-rich solution during seed inhibition is viable and potentially scalable to nursery production. Similarly, the approach of bathing seedling root systems in a nutrient-rich endophyte solution prior to transplanting them into field conditions highlights the potential of liquid consortia application for future work at scales relevant to forestry plantations and wildland restoration efforts.

Endophyte inoculated seedlings had higher survival than control seedlings under the environmental stress conditions imposed during experimentation. The experimental conditions in the fertilized and unfertilized conditions showed no difference in survival, presumably because the soil mixture was not stressful for initial establishment. Seedlings of both species inoculated with the Salicaceae family strains comprising Consortium 1+2 had a substantially improved survival rate following two growing seasons of drought simulation (Tables 3, 4). Both species showed a subtle but positive trend following inoculation, despite challenging edaphic factors and stressful climatic conditions of field trials. Soil volumetric water content was ≤ 10% as early as April 2014, only a week after transplant of the *P. menziesii*, and prior to bud break of the *T. plicata* seedlings. Then several heat waves extended regional drought in 2015 (Fosu et al., 2016). *T. plicata* survival rates at the field site suggest Consortium 3, which included all the conifer endophyte strains, was the most effective with individuals persisting through the final measurement date (Table 2). The *P. menziesii* survival rate was low (<20%) soon after transplant. Of note was a 10% survival rate among the *P. menziesii* seedlings that were treated with Consortium 1+2 (Khan et al., 2016); no control seedlings survived to final measurement. *Phellinus* spp., apparent on residual mature *P. menziesii* stumps, provided additional seedling stress, and although not necessarily lethal to young *P. menziesii* in combination with other stressors, seedlings may have been more susceptible to root loss and subsequent desiccation from infection (Rodriguez et al., 2009).

More than a year was necessary for inoculation to benefit seedling performance. Following approximately 360 DAI, both species maintained better metabolic function (i.e., *F*_v_/*F*_m_ and SPAD) in the presence of moisture stress or despite nutrient deficit, respectively. Previous work demonstrated a similar lag time in colonization of bacterial endophytes in *P. menziesii* and other tree species (Parker and Dangerfield, 1975; Khan et al., 2015) and in rice (Kandel et al., 2017b). A review of temporal and spatial infection patterns of endophytes suggests seasonal variation, favorable growing conditions (e.g., temperature and humidity), and host genotype can influence rate and extent of colonization (Wilson, 2000). Furthermore, *P. menziesii*, *T. plicata*, *Pinus contorta*, and *Populus trichocarpa*, inoculation with a single diazotrophic bacterial strain resulted in negative or lag effects which persist for 9–13 months (Parker and Dangerfield, 1975; Anand and Chanway, 2013; Anand et al., 2013; Knoth et al., 2014).

Inoculation of seed during inhibition mitigated the lag effect. Chanway et al. (2000) demonstrated successful inoculation of conifer seed with PGPR and subsequent benefits to seedling growth. In another study, colonization of rhizodermal tissue by PGPR led to subsequent colonization of stem and leaf tissues through the transpiration stream from root xylem vessels (Compant et al., 2005). The *P. menziesii* seedlings showed an increase in radicle length for fertilized and inoculated approximately a month after sowing (∼50 DAI; Table 5); and at final harvest both branching and RDM were higher than among un-inoculated control seedlings. This germinant radicle elongation phenomenon was described by Glick et al. (1997), where PGPR inoculation directly to the seed coat lowered ethylene concentrations (hydrolyzing of ACC deaminase), which in turn lowers the ethylene caused inhibition of root elongation following germination. Increased branching among *P. menziesii* seedlings – even in the absence of fertilizer – was notable (Table 6), and likely a function of PGPR phytohormone. Many endophytes, including genus *Burkholderia*, demonstrate both ACC and IAA synthesis, which are linked with suppressing stress-induced ethylene production and with increased formation of branches, and lateral and adventitious roots, respectively (Chanway et al., 1991; Khan et al., 2016; Santoyo et al., 2016).

The positive effects of endophyte inoculation on conifer growth developed slowly and varied by experiment. The Salicaceae endophyte strains used in Experiment 2 showed a limited net-positive response from inoculation, but only after ∼360 DAI for *P. menziesii*. A decrease in mean RCD growth occurred in both species and was expectedly pronounced among seedlings from the driest moisture regime. When root water uptake is unable to meet transpiration, a negative water balance leads to lost carbohydrate reserves during root egress, and systematic desiccation; both potentially cause reductions in RCD (Burdett, 1990; Davis and Jacobs, 2005; Grossnickle, 2012; Villar-Salvador et al., 2015). Limited height growth among the inoculated seedlings was also observed following the first drought period (Figure 1 and Tables 3, 4). *P. menziesii* seedlings subject to the drier and wetter moisture regimes had more biomass at the final harvest with inoculation. *T. plicata* seedlings assimilated the multi-strain consortia with limited negative growth response following the initial pronounced drought simulation. Drier conditions did limit RCD growth for *T. plicata* < 360 DAI, but showed no clear effect on height or RCD resulting from inoculation treatments. Similar to *P. menziesii*, inoculated *T. plicata* seedlings’ RDM and SDM were indistinguishable from the control; an increase in biomass among inoculated seedlings correlated to increased moisture availability (wetter treatment conditions). Although not statistically significant, these trends are consistent with previous research findings with the same species (Anand and Chanway, 2013; Khan et al., 2015).

Predictably, fertilized plants yielded larger and healthier seedlings of both species. However, seedling growth was positively influenced by conifer endophyte inoculation, regardless of the presence of fertilizer. The increase in rooting potential is the most striking finding. Inoculated *P. menziesii* seedlings had an advantage during taproot egress (Table 5) following germination, when fertilizer in the rhizosphere has negligible influence. Both species later developed more fibrous root (i.e., >FOLR) systems if they were inoculated with conifer endophytes, with *T. plicata* also having more root biomass (Table 6). Increased presence of endophytes in rhizodermal tissues and subsequent increases in rooting capacity of plants following inoculation has previously been reported (Compant et al., 2005; Khan and Doty, 2009). Four of the six conifer endophyte strains produced the hormone, indole-3-acetic acid (IAA), known to affect host rooting (Sukumar et al., 2013).

Salicaceae endophyte strains and many of the conifer endophyte strains used in our evaluation including WP1, PTD1, WP19, WP5, WP9, WW5, WW6, WW7 (Table 1), have been previously characterized (Khan et al., 2016) and were shown to produce IAA, abscisic acid (ABA), gibberellic acid (GA), brassinosteroids (BR), jasmonates (JA), and salicylic acid (SA). The aforementioned phytohormones are stress responsive and can influence seedling root and shoot morphology (Ryan et al., 2008; Santoyo et al., 2016). Work with the same endophyte strains showed mutualistic benefits, specifically biomass and root growth improvements in *Zea mays* (Knoth et al., 2013), *P. menziesii* (Khan et al., 2015), and *Populus* spp. (Knoth et al., 2014; Khan et al., 2016). Robust root development increases the quality of planting stock (Davis and Jacobs, 2005), and advantages to post-transplant root egress can improve survival and early development (Grossnickle, 2012).

The plant survival, physiology, and morphological growth trends during the first years of establishment provide insight into longer-term growth trajectory and development. Previous work with hybrid poplar (Khan and Doty, 2009; Khan et al., 2012, 2016; Knoth et al., 2014) and *P. menziesii* (Khan et al., 2015) employed multi-strain consortia to elicit benefits to developing seedlings. Those trials demonstrated inoculation improved biomass, stress mitigation, and phenotypic plasticity among targeted tree species. Working with a consortium of the same Salicaceae strains employed in our experiments, Rho et al. (2018b) found that inoculation of rice led to significant increases in cumulative water use efficiency that was linked with increased leaf-level ABA production and increased stomatal control, without deleterious effects to biomass accumulation. Building on this, our work shows inoculation confers benefit to the function of PSII and mechanisms for chlorophyll production (i.e., chlorophyll fluorescence), and measurable differences were detected following 1 year of endophyte residency within seedlings.

Chlorophyll fluorescence is a meaningful indicator of environmental stressors including drought stress, sun/shade needle effects, and even seasonal stress variation (Mohammed et al., 1995). As our measurements progressed through simulation of summer drought (Figure 2) in Experiment 2, higher values persisted among the Salicaceae endophyte-inoculated seedlings of both species in the second growing season (>365 DAI). Specifically, the inoculated treatment groups often maintained values between 0.79 and 0.83 later into the increased period of rhizosphere moisture deficit. A similar measurable difference between inoculated and control seedlings was measured in *Populus* spp. seedlings less than 90 DAI and 19 days after onset of drought stress (Khan et al., 2016). From Experiment 3, only *T. plicata* demonstrated a measurable significant difference in *F*_v_/*F*_m_ to fertilizer and conifer endophyte inoculation treatments (Figure 5), and also in the second growing season as CRF presence in the rhizosphere dissipated. An interaction effect between endophytes and water treatment appears for both species in the drought experiment at the height of the growing season, and similarly for just *T. plicata* in the fertilization experiment. In both cases, the height of the growing season is when there is a direct (experimentally induced) or indirect (ambient temperature induced) onset of moisture stress, which is likely when the endophytes have the most meaningful (i.e., measurable) impact on plant physiological status. This consideration merits future investigations into the specific mechanisms of the effects in future work with *F*_v_/*F*_m_ and other relevant physiological metrics. Values ≥0.83 are considered common for non-stressed leaves (Baker, 2008; Cessna et al., 2010). The un-inoculated control groups’ values eventually fell below 0.79, a threshold for a well-functioning photosynthetic apparatus that was derived from a limited sample of conifers (Fang-yuan and Guy, 2004). Past research monitoring a broader group of temperate conifers’ responding to stressors including light/shade, moisture, and temperature shows that undamaged seedling *F*_v_/*F*_m_ values ranged significantly from 0.5 to 0.83 (Khan et al., 2000; Fang-yuan and Guy, 2004; Ritchie, 2006). Our measurements of chlorophyll fluorescence are best interpreted as relative differences in performance between treatment groups and should serve as baseline for future evaluations of these species or congeners.

Shifts in SPAD values during the second year of Experiment 2 (Figure 3) suggest Salicaceae endophyte inoculation-maintained chlorophyll content in *P. menziesii* and *T. plicata* leaf tissue during increasing moisture deficit. Increased SPAD values were noted among *Populus* spp. seedlings inoculated with multi-strain mixtures compared to single strain and control groups (Knoth et al., 2014). Inoculating with multi-strain consortia also increased nitrogen content in *P. menziesii* (Khan et al., 2015) and demonstrated efficacy in reducing degradation of chlorophyll content in *Populus* spp. in experimental drought scenarios. Increased chlorophyll content of both species’ seedlings persists among the conifer endophyte inoculated group and is catalyzed by the presence of fertilizer (Figures 4, 6). The observed delays in degradation of chlorophyll could be attributed to nitrogen fixing capability, phosphate solubilization, and iron chelating traits of strains in both experiments. Two of the conifer endophyte strains, TPSK3 and TPSN7, demonstrated N-fixation capability in the culture conditions of the ARA. In addition, strains TPSK5, PMPF3, and PMSK6 contained the nitrogenase subunit gene, and, therefore, may have the capacity to fix nitrogen. Increased availability of nitrogen and phosphorus can contribute to healthy development of leaf tissue necessary for the photosynthetic apparatus, and increased iron availability can improve plant stress tolerance (Aroca, 2012).

The *P. menziesii* results in the drought experiment are particularly interesting, despite confounding introduced by an unexpected outbreak of host-specific black pineleaf scale (*Nuculaspis californica*) which began ∼6 months before the end of the trial (∼365 DAI). Measurement of leaf tissue (2 cm^2^ from *N* = 5/trt) determined the invasion was uniform across all treatment groups; therefore, no measures were taken to control the outbreak. The results for *P. menziesii* are consistent with the added stressor of this insect to an unknown degree. The production of toxic alkaloids, the likely mediators of insect resistance, can be enhanced by endophytes (Arnold et al., 2003; Ganley et al., 2008; Miller, 2011) and increased levels can be triggered by abiotic stressors like drought (Bultman and Bell, 2003; Findlay et al., 2003; Eyles et al., 2010). The improved survival rates among drought stressed inoculated *P. menziesii* seedlings during Experiment 2, and subject to the otherwise lethal insect invasion, merits investigation into the insect resistant mutualistic properties of these strains or the consortium.

Our field trials demonstrated that extreme field conditions may mask immediate beneficial effects derived from endophytes applied into the rhizosphere following transplant. Under more controlled conditions, seedlings of both species displayed net positive growth and metabolic effects when the stress induced was relatively low (i.e., moisture availability higher or fertilizer present). This suggests field trials on more mesic site conditions may yield a beneficial effect on the host conifers featured in our research. Further, the carbon cost of establishing endophytic relationships with the plant host may be responsible for reduced plant growth before ∼365 DAI (Knoth et al., 2014), but lag time may be mitigated by inoculating seed. The beneficial nature of individual strains is likely host-species specific; however, we demonstrate that a multi-strain approach may be more efficient in garnering a positive colonization effect and growth response.

## Data Availability

All datasets generated for this study are included in the manuscript and/or the supplementary files.

## Author Contributions

MA, ZK, and GE conceived and designed the experiments. MA, ZK, MJ, AMS, and AWS performed the experiments. MA, ZK, MJ, GE, AMS, AWS, and SD analyzed the data. SD and GE contributed reagents, materials, and analysis tools. MA, ZK, MJ, GE, and SD wrote the manuscript.

## Conflict of Interest Statement

The authors declare that the research was conducted in the absence of any commercial or financial relationships that could be construed as a potential conflict of interest.
